# Cardiovascular magnetic resonance of the myocardium at risk in acute reperfused myocardial infarction: comparison of T2-weighted imaging versus the circumferential endocardial extent of late gadolinium enhancement with transmural projection

**DOI:** 10.1186/1532-429X-12-18

**Published:** 2010-03-29

**Authors:** Joey FA Ubachs, Henrik Engblom, David Erlinge, Stefan Jovinge, Erik Hedström, Marcus Carlsson, Håkan Arheden

**Affiliations:** 1 Department of Clinical Physiology, Lund University and Skåne University Hospital, Lund, Sweden; 2 Department of Cardiology, Lund University and Skåne University Hospital, Lund, Sweden

## Abstract

**Background:**

In the situation of acute coronary occlusion, the myocardium supplied by the occluded vessel is subject to ischemia and is referred to as the myocardium at risk (MaR). Single photon emission computed tomography has previously been used for quantitative assessment of the MaR. It is, however, associated with considerable logistic challenges for employment in clinical routine. Recently, T2-weighted cardiovascular magnetic resonance (CMR) has been introduced as a new method for assessing MaR several days after the acute event. Furthermore, it has been suggested that the endocardial extent of infarction as assessed by late gadolinium enhanced (LGE) CMR can also be used to quantify the MaR. Hence, we sought to assess the ability of endocardial extent of infarction by LGE CMR to predict MaR as compared to T2-weighted imaging.

**Methods:**

Thirty-seven patients with early reperfused first-time ST-segment elevation myocardial infarction underwent CMR imaging within the first week after percutaneous coronary intervention. The ability of endocardial extent of infarction by LGE CMR to assess MaR was evaluated using T2-weighted imaging as the reference method.

**Results:**

MaR determined with T2-weighted imaging (34 ± 10%) was significantly higher (p < 0.001) compared to the MaR determined with endocardial extent of infarction (23 ± 12%). There was a weak correlation between the two methods (r^2 ^= 0.17, p = 0.002) with a bias of -11 ± 12%. Myocardial salvage determined with T2-weighted imaging (58 ± 22%) was significantly higher (p < 0.001) compared to myocardial salvage determined with endocardial extent of infarction (45 ± 23%). No MaR could be determined by endocardial extent of infarction in two patients with aborted myocardial infarction.

**Conclusions:**

This study demonstrated that the endocardial extent of infarction as assessed by LGE CMR underestimates MaR in comparison to T2-weighted imaging, especially in patients with early reperfusion and aborted myocardial infarction.

## Background

The myocardium at risk (MaR), defined as the hypoperfused myocardium during acute coronary occlusion will be subject to infarction if no reperfusion occurs[1]. The ability to assess MaR in relation to the final infarct size enables determination of myocardial salvage and, consequently, the efficacy of reperfusion therapy in patients with acute coronary occlusion [2,3].

Currently, the most widely used method to determine myocardial perfusion defects is myocardial perfusion single photon emission computed tomography (SPECT). This technique can be used to assess MaR in the acute setting by intravenously injecting a perfusion tracer during coronary occlusion [4,5]. However, this approach has limitations in the clinical setting since the patient needs to have the perfusion tracer injected prior to reperfusion and undergo imaging in a gamma camera within approximately 3 hours. Thus, using myocardial perfusion SPECT for determination of MaR in patients with acute coronary occlusion is a major logistic challenge not possible at many hospitals[6]. Hence, there is a need for more clinically feasible methods to assess MaR that can be performed after the acute revascularization.

T2-weighted cardiovascular magnetic resonance (CMR) has recently been introduced as a method for quantification of MaR, and has been validated in both animals[7] and humans[8]. Furthermore, it has recently been suggested that the endocardial extent of infarction as assessed by late gadolinium enhanced (LGE) CMR can be used to determine MaR [9,10]. The pathophysiological basis for using endocardial extent of infarction as a measure of MaR is that previous experimental studies have shown that the endocardial extent of infarction is established approximately 40 minutes after coronary occlusion[11]. Thereafter, the infarcted area will increase by transmural progression from the endocardium to the epicardium with increasing duration of ischemia, referred to as the wavefront phenomenon[11]. Thus, time to reperfusion is thought to limit the transmural infarct progression rather than the endocardial extent of infarction. This implicates that the endocardial extent of infarction could potentially be used for assessing the MaR after acute coronary occlusion. However, in the situation of early reperfusion, infarction might be completely or almost completely aborted [12,13], resulting in difficulties when assessing MaR based on infarct characteristics.

Therefore, we sought to assess the ability of endocardial extent of infarction assessed by LGE CMR to predict MaR as compared to T2-weighted imaging in patients with first-time early reperfused myocardial infarction.

## Methods

### Study population and design

The study was approved by the local ethics committee and all patients gave their written informed consent. Thirty-seven patients (age; 62 ± 10, 32 males) with first-time myocardial infarction, presenting with acute ST-elevation myocardial infarction (STEMI), due to a single occluded coronary artery as seen by angiography, were included in the study. Patients with a non-occluded culprit artery, contraindications for CMR such as metal implants, signs of an old infarction were excluded. All patients were treated by primary percutaneous coronary intervention (PCI) with coronary stenting, resulting in TIMI grade 3 flow in the culprit artery. CMR was performed within the first week after PCI in all patients.

### CMR

CMR was performed on either of two 1.5-T systems: Magnetom Vision (Siemens, Erlangen, Germany) with a CP body array coil, or Philips Intera CV (Philips, Best, the Netherlands) with a cardiac synergy coil. All subjects were placed in supine position and images were acquired at end-expiratory breath hold with electrocardiographic gating. Initial scout images were acquired to locate the heart, and a T2-weighted triple inversion turbo spin echo sequence was employed to depict the myocardium at risk. T2-weighted images were acquired in the short-axis view, covering the left ventricle from base to apex. Imaging parameters for the T2-weighted sequence were: echo time, 43 ms (Siemens), or 100 ms (Philips); repetition time, 2 heart beats; number of averages, 2; inversion time, 180 ms; image resolution, 1.5 × 1.5 mm; slice thickness, 10 mm (Siemens), or 8 mm with a slice gap of 2 mm (Philips). When acquiring images with the cardiac synergy coil, parallel imaging with SENSE = 1 was used to minimize signal inhomogeneities due to differences in coil sensitivity.

Long- and short-axis late gadolinium enhanced (LGE) images covering the entire left ventricle were then acquired approximately 20 minutes after intravenous administration of 0.2 mmol/kg extracellular gadolinium-based contrast agent (gadoteric acid; Guerbet, Gothia Medical AB, Billdal, Sweden). The LGE images were acquired with an inversion-recovery sequence with following imaging parameters; slice thickness 10 mm, field of view 380 mm, matrix 126 × 256, flip angle 25°, repetition time 100 ms, echo time 4.8 ms (Siemens) or slice thickness 8 mm, field of view 340 mm, repetition time 3.14 ms, echo time 1.58 ms (Philips). Inversion time was adjusted to null the signal from viable myocardium[14].

### Image analysis

All CMR images were analyzed using the freely available software Segment v1.8 http://segment.heiberg.se[15]. The T2-weighted MaR was identified according to a previously described methodology[8]. In short, in all short-axis slices, the hyperintense regions were delineated manually by independent and blinded observers after manual tracing of the endocardial and epicardial borders of the left ventricle. The papillary muscles were excluded from the myocardium. The MaR was then defined as the total amount of hyperintense myocardium in all short-axis slices and expressed as percentage of left ventricular myocardium. If present, an area of hypointense signal within the area of increased signal intensity was included in the MaR.

The infarcted myocardium was automatically quantified from the short-axis LGE images according to a previously described method[15]. In short, the endocardial and epicardial borders were traced manually with exclusion of the papillary muscles. The LGE myocardium was then defined using a computer algorithm that takes into consideration partial volume effects within the infarcted region. Manual adjustments were made when the computer algorithm was obviously wrong. If present, a hypointense signal within the area of LGE (microvascular obstruction) was included in the analysis as being completely infarcted. Finally, myocardial infarct size was expressed as percent of left ventricular myocardium.

**Figure 1:**
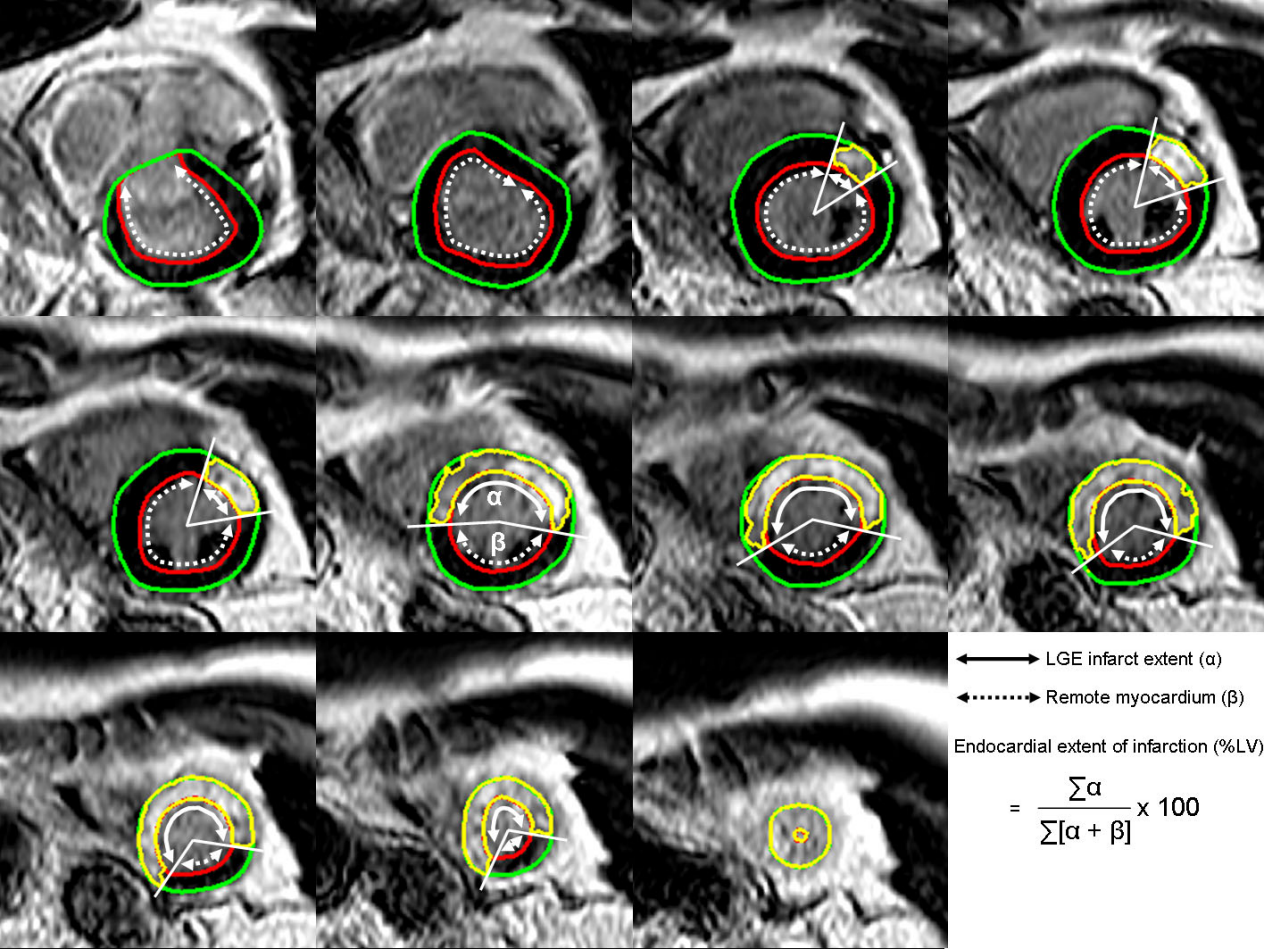
**Endocardial extent of infarction**. Late gadolinium enhanced (LGE) CMR showing short-axis slices covering base (top left) to apex (bottom right) in a patient with an occlusion of the left anterior descending coronary artery. The endocardial borders are traced in red and the epicardial borders are traced in green. The infarct is shown as an LGE area within the myocardial borders and is delineated in yellow. The circumferential endocardial extent of LGE is marked by the solid arrow (α) and the remote myocardium is marked by the dashed arrow (β). The endocardial extent of MI was defined as the sum of the LGE circumferential endocardial extent in each short-axis slice (∑α), divided by the total endocardial extent (∑ [α + β]) of the LV. In this patient, the MaR by T2-weighted imaging and LGE endocardial extent was 37% and 32%, respectively. The corresponding infarct size was 32%, resulting in a myocardial salvage of 13% and 1% when measured with T2-weighted imaging and LGE endocardial extent, respectively.

The myocardium at risk by endocardial extent of infarction was determined in each short-axis slice by measuring the circumferential distance between the lateral borders of the LGE region (Figure 1). The lateral borders of LGE were used since the mid-mural LGE might extent beyond the endocardial circumferential edges. The endocardial extent of MI was then defined as the sum of the LGE endocardial circumferential distance in each short-axis slice, divided by the total endocardial extent of the LV.

Myocardial salvage was defined as 100*([MaR - infarct size]/MaR), where MaR was assessed by both T2-weighted imaging and endocardial extent of infarction.

### Statistical methods

Continuous variables are presented as mean ± SD. Pearson's correlation was used to determine the relationship between MaR assessed by T2-weighted imaging and endocardial extent of infarction. The agreement between T2-weigthed imaging and endocardial extent of infarction was expressed as mean difference ± SD, and the limits of agreement were shown in a Bland-Altman graph as mean ± 2 SD. The difference between using T2-weighted imaging and endocardial extent of infarction for determination of MaR to derive myocardial salvage, was assessed by a paired t-test. SPSS version 17.0 software package (Chicago, Illinois, USA) was used for analysis. A value of p below 0.05 was considered statistically significant.

## Results

The characteristics of the 37 patients included in this study are presented in Table 1. In 59% (22/37) of all patients, the left anterior descending coronary artery was the culprit vessel and in 35% (13/37) of the patients, the right coronary artery was the culprit vessel. All patients underwent CMR imaging within seven days (mean 4 ± 3 days) after percutaneous coronary intervention.

**Table 1:** Characteristics

Male	32	86%
Age (y)	62 ± 10	(36 - 83)
Time from pain onset to reperfusion	200 ± 132	(80 - 565)
Time from PCI to CMR (days)	4 ± 3	(0 - 7)
Occluded artery by angiography		
LAD	22	59%
RCA	13	35%
LCX	1	3%
Left Main	1	3%
Infarct Size (% of LV myocardium)	14 ± 10	(0 - 47)
Endocardial Extent (% of LV myocardium)	23 ± 12	(0 - 66)
MaR by T2-weighted imaging (% of LV myocardium)	34 ± 10	(16 - 59)
Myocardial Salvage by T2-weighted imaging (% salvage of MaR)	58 ± 22	(13 - 100)
Myocardial Salvage by endocardial extent (% salvage of MaR)	45 ± 23	(-7 - 100)

Values are presented as n (%) or as mean + SD (range). PCI = percutaneous coronary intervention; CMR = cardiovascular magnetic resonance; LAD = left anterior descending artery; RCA = right coronary artery; LCx = left circumflex artery; MaR = myocardium at risk.

### Myocardium at risk

A region with increased signal intensity by T2-weighted imaging was observed in all patients, resulting in a myocardium at risk of 34 ± 10% (range 18 - 59) of the left ventricular (LV) myocardium. The endocardial extent of infarction was 23 ± 12% (range 0 - 66) of the total LV endocardial surface.

**Figure 2:**
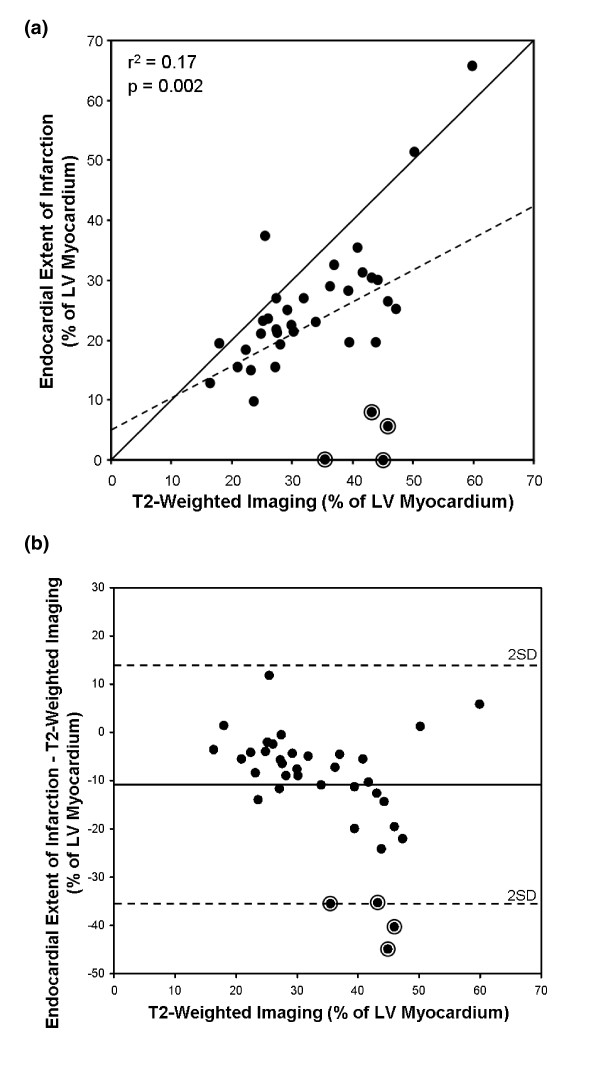
**Myocardium at risk by T2-weighted imaging and by endocardial extent of infarction**. A) MaR by endocardial extent of infarction versus T2-weighted imaging. Solid line = line of identity; dashed line = regression line; r^2 ^= 0.17, p = 0.002. The two patients with an aborted infarction and the two patients with more than 90% myocardial salvage are encircled. B) Bland-Altman graph showing the difference between myocardium at risk quantified by endocardial extent of infarction versus T2-weighted imaging. The difference between endocardial extent of infarction and T2-weighted imaging was -11 ± 12%. Solid line = mean of endocardial extent of infarction - T2-weighted imaging; dashed lines = ± 2 SD. The two patients with an aborted infarction and the two patients with more than 90% myocardial salvage are encircled.

Figure 2A shows a scatter plot indicating the relationship between T2-weighted imaging and endocardial extent of infarction. There was a weak correlation between the two methods (r^2 ^= 0.17, p = 0.002). Figure 2B shows the limits of agreement between endocardial extent of infarction and T2-weighted imaging, demonstrating an underestimation of the myocardium at risk by endocardial extent of infarction (mean difference -11 ± 12%). Two patients showed no signs of infarction by LGE CMR and two patients showed > 90% salvage (encircled in Figure 2A and Figure 2B). All four patients had, however, an occluded artery on angiography, ST-elevation indicative of transmural ischemia and biomarker release indicative of myocyte necrosis. Myocardium at risk in these four patients measured 36 - 46% by T2-weighted imaging and 0 - 8% by endocardial extent of infarction.

**Figure 3:**
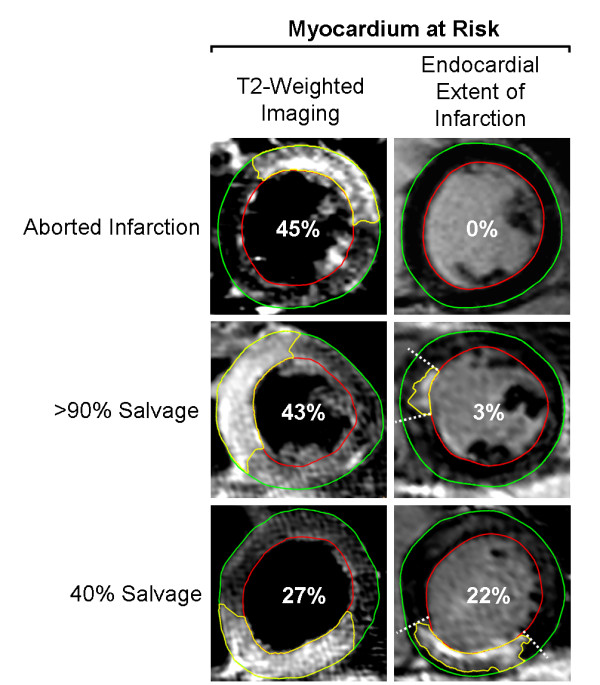
**Myocardium at risk by T2-weighted imaging and endocardial extent of infarction**. Short-axis slices at the same ventricular level of T2-weighted imaging and LGE CMR for endocardial extent of infarction in 3 patients after reperfusion of an acute coronary occlusion. The endocardial borders are traced in red, the epicardial borders are traced in green and the affected region is traced in yellow (MaR for T2-weighted imaging and infarction for LGE CMR). The borders of the endocardial extent of infarction are indicated by dashed lines. Within each image the total MaR is given as a percent of left ventricle. The upper panel shows a patient with an aborted infarction, the middle panel a patient with > 90% myocardial salvage and the lower panel a patient with 40% myocardial salvage. Note the difference in size of the MaR by T2-weighted imaging and endocardial extent of infarction for the patient with an aborted infarction and the patient with > 90% myocardial salvage.

Figure 3 shows three examples of the agreement of MaR by T2-weighted imaging and endocardial extent of infarction by LGE CMR.

There was no statistical significant correlation between MaR measured by T2-weighted imaging or endocardial extent of infarction and time from pain onset to reperfusion (Table 2).

**Table 2:** The relationships between CMR findings and clinical characteristics

	Time to PCI*		CK-MB max.		Troponin T max.	
	**R**^2^	p	**R**^2^	p	**R**^2^	p
T2-weighted imaging	0.04	0.23	0.05	0.22	0.07	0.13
LGE endocardial extent	0.0001	0.80	0.36	< 0.01	0.29	< 0.01
LGE infarct size	0.002	0.96	0.37	< 0.01	0.33	< 0.01
Salvage by T2-weighted imaging	0.008	0.62	0.26	< 0.01	0.20	< 0.01
Salvage by LGE endocardial extent	0.0004	0.92	0.25	< 0.01	0.19	0.01

* Time from pain onset to reperfusion. PCI = percutaneous coronary intervention. LGE = late gadolinium enhancement.

### Myocardial infarction

**Figure 4:**
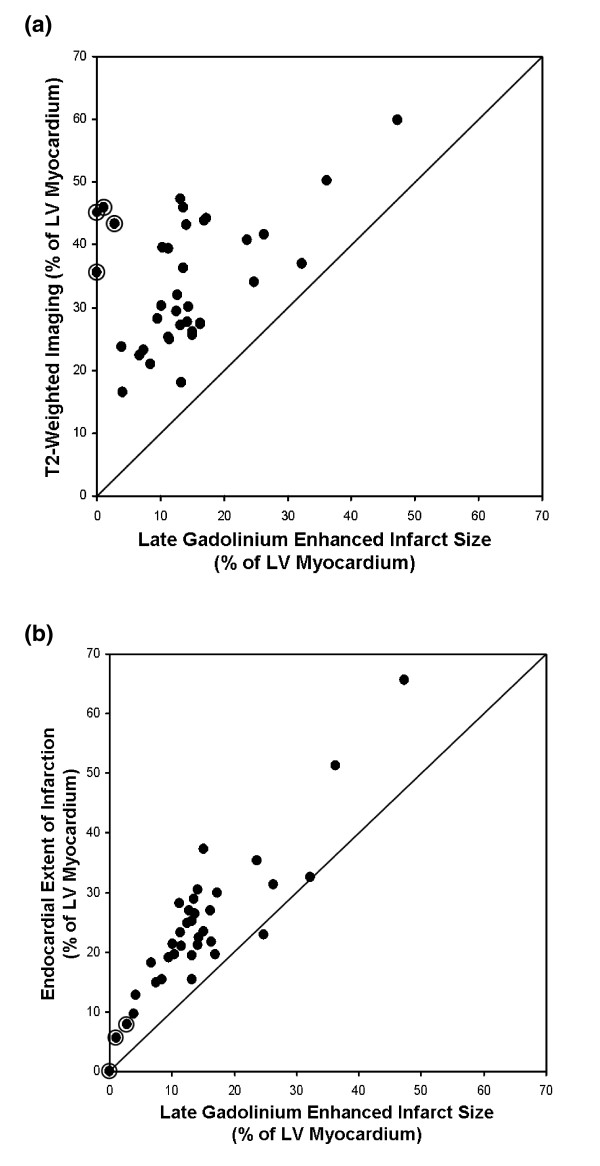
**Myocardium at risk by T2-weighted imaging and endocardial extent of infarction versus infarct size by late gadolinium enhanced CMR**. A) Myocardium at risk by T2-weighted imaging in relation to infarct size by late gadolinium enhanced CMR presented with the line of identity. The myocardium at risk by T2-weighted imaging was consistently greater than the infarct size in all patients. B) Myocardium at risk by endocardial extent of infarction in relation to infarct size by late gadolinium enhanced CMR presented with the line of identity. The myocardium at risk by endocardial extent of infarction was, in general, greater than the infarct size. For one patient the infarct size was, however, greater than the myocardium at risk. The two patients with an aborted infarction and the two patients with more than 90% myocardial salvage are encircled.

The infarct size by LGE CMR was 14 ± 10% (range 0 - 47) of the LV myocardium. In comparison to the MaR by T2-weighted imaging, the infarct size was smaller in all patients (p = 0.002) (Figure 4A). For one patient the MI size was greater than the MaR as assessed by endocardial extent of infarction (Figure 4B).

The infarct size significantly correlated with peak CK-MB and peak Troponin T. There was, however, no correlation between infarct size and time from pain onset to reperfusion (Table 2).

### Myocardial salvage

**Figure 5:**
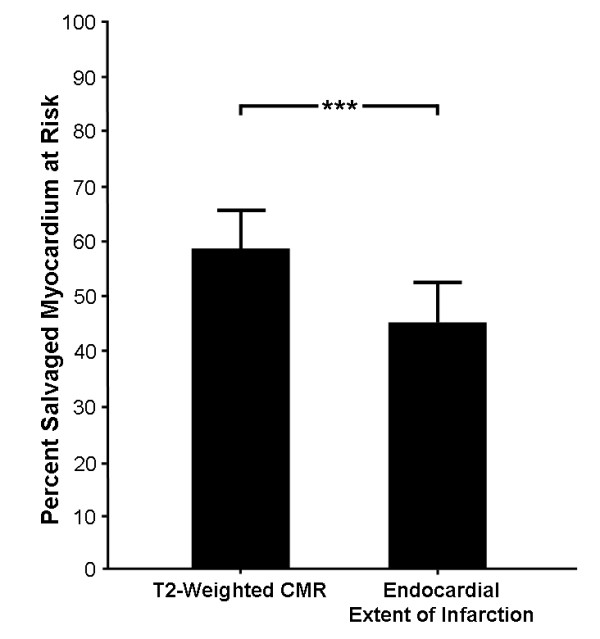
**Myocardial Salvage**. Mean percent myocardial salvage with T2-weighted imaging and endocardial extent of infarction as the measure for MaR. Myocardial salvage assessed with T2-weighted imaging was significantly higher than when assessed with endocardial extent of infarction. Error bars indicate two standard error of the means.

Comparison of the infarct size by LGE CMR with the MaR by T2-weighted imaging and endocardial extent of infarction yielded a myocardial salvage of 58 ± 22% (range 13 - 100) and 45 ± 23% (range -7 - 100), respectively (Figure 5). Thus, myocardial salvage assessed by T2-weighted imaging was significantly higher than when assessed with endocardial extent of infarction (p < 0.001).

There was not a significant correlation between myocardial salvage, measured by T2-weighted imaging and endocardial extent of infarction, and time from pain onset to reperfusion (Table 2).

## Discussion

This study shows that endocardial extent of infarction as assessed by LGE CMR underestimated the myocardium at risk in comparison to T2-weighted imaging in patients undergoing early reperfusion therapy of first-time myocardial infarction.

In the experimental setting, it has been shown that approximately 40 minutes after coronary occlusion the extent of endocardial necrosis is established and remains equal up to 8 weeks after the acute coronary occlusion [11,16]. When the endocardial extent is established, the infarction will progress from the endocardium to the epicardium as the duration of ischemia increases. This is often referred to as the wavefront phenomenon[11]. Thus, time to reperfusion is thought to limit the transmural infarct progression rather than the endocardial extent of the infarct.

In the present study, the endocardial extent of infarction significantly underestimated the MaR compared to T2-weighted imaging. This is in accordance with recent findings by Wright et al[10], where a difference between the two methods was found in patients where pronounced myocardial salvage or infarct abortion was achieved with acute reperfusion therapy. Some patients in the present study showed no signs of infarction by LGE CMR but did, however, show evidence of ischemic myocardium by T2-weighted imaging, as was also seen by Wright et al[10]. The correlation between endocardial extent of infarction and MaR by T2-weighted imaging was, however, significantly weaker in the present study compared to their study (r^2 ^= 0.17 vs r^2 ^= 0.59). This difference may be due to fewer patients and a limited range of MaR by T2-weighted imaging in the present study.

When using endocardial extent of infarction for the assessment of MaR, several aspects need to be considered. Following acute coronary occlusion, myocytes swell due to failure of energy-regulated membrane channels, resulting in intracellular influx of water and sodium[17]. If ischemia persists, the cell membrane will disintegrate, which is the onset of necrosis, resulting in increased distribution volume for the gadolinium-based contrast agent used for infarct visualization[18]. Recently, Cury et al[19] have shown that an increased signal on T2-weighted images can be found in the absence of LGE in patients presenting with clinical signs of acute coronary syndrome. Furthermore, Abdel-Aty et al[20] found that there was an apparent change in signal intensity on the T2-weighted images at a mean of 28 minutes after coronary occlusion in dogs. However, despite an increased signal on the T2-weighted images present after approximately 30 minutes, no LGE was found in the ischemic myocardium. Thus, if early reperfusion is accomplished in the situation of acute coronary occlusion, there might be an absence of LGE on the CMR images, referred to as aborted infarction [12,13]. Consequently, LGE CMR imaging does not allow for determination of MaR in this situation, whereas T2-weighted imaging does. The present study showed a significant difference between T2-weighted imaging and endocardial extent of infarction for the quantification of MaR. This can in part be explained by the presence of an aborted infarction. The current study had two patients with an aborted infarction and two patients with a myocardial salvage of more than 90% of the initial myocardium at risk as assessed by T2-weighted imaging. In these four patients, the MaR was 36 - 46% of the LV as assessed by T2-weighted imaging and 0 - 8% as assessed by endocardial extent of infarction, affecting the determination of myocardial salvage in the patients significantly.

In an experimental setting, Fieno et al showed that the endocardial extent of infarction might change over time after infarction as assessed by LGE CMR[16]. Furthermore, Engblom et al[21] recently showed, in a human population, that the endocardial extent of LGE significantly decreased during the first week after coronary occlusion. This was explained by a possible decrease in LGE of a viable peri-infarction zone that surrounded the irreversibly injured core of myocytes in the early post-infarction period. This reduction of endocardial LGE implicates a difference in MaR between day one and week one when assessed by CMR. Thus, the endocardial extent of infarction as measured with LGE CMR may change within the first week after coronary occlusion due to initial enhancement of the peri-infarction zone not present at one week. Myocardium at risk by T2-weighted imaging, however, does not change during the first week after infarction[8].

T2-weighted imaging has been shown to enable distinction between acute and chronic myocardial infarction[22]. Hence, MaR can potentially be assessed even in the presence of old infarction. Presence of old infarction, however, disables determination of MaR using endocardial extent of infarction by LGE CMR.

Another situation where MaR assessed by endocardial extent of infarction might be misleading is in the case of periprocedural induction of MI, which has been reported to occur in approximately 6% of all patients with non-STEMI undergoing acute reperfusion therapy[23]. A significant myocardial salvage might be missed if MaR is based on the endocardial extent of a small transmural PCI-induced infarct caused by distal embolization of a small coronary branch.

In the present study, no significant correlation was found between myocardial salvage and time from pain onset to reperfusion. The relationship between myocardial salvage and duration of ischemia in humans has recently been described in a study by Hedström et al[3]. The inclusion and exclusion criteria in the present study differ significantly from those applied by Hedström et al. For example, patients with biomarker release prior to PCI were excluded in the latter. The present study, however, included a more general population presenting with clinical signs of acute myocardial infarction. Thus, some patients in the present study had biomarker release prior to PCI, which may be an indicator of spontaneous reperfusion and re-occlusion that has been shown to sometimes occur in the early phase of acute coronary thrombosis[24]. As a consequence, the duration of persistent ischemia may be overestimated in these patients, affecting the relationship between myocardial salvage and duration of ischemia assessed in the present study.

### Limitations

The present study was performed on a patient population presenting with first-time STEMI and undergoing successful reperfusion therapy. Thus, the applicability of the current results to settings like non-reperfused acute MI remains illusive. Furthermore, since only five women were included in the study the results cannot be generalized to both genders.

No independent measure of MaR, such as myocardial perfusion single photon emission computed tomography (SPECT) was obtained in the present study. Previous studies, however, have shown that T2-weighted imaging agrees well with myocardial perfusion SPECT [3,8].

Image quality can be a limitation in assessing MaR by T2-weighted imaging, and the image quality of the T2-weighted images used in the present study did not allow for automatic segmentation[8].

## Conclusions

This study demonstrated that the endocardial extent of infarction as assessed by LGE CMR underestimates the myocardium at risk in comparison to T2-weighted imaging, especially in patients with early reperfusion and aborted myocardial infarction.

## Abbreviations

CMR: Cardiovascular Magnetic Resonance; LGE: Late Gadolinium Enhanced; LV: Left Ventricle; MaR: Myocardium at Risk; STEMI: ST-Elevation Myocardial Infarction; SPECT: Single Photon Emission Computed Tomography; T: Tesla; TIMI: Thrombolysis in Myocardial Infarction.

## Competing interests

The authors declare that they have no competing interests.

## Authors' contributions

JU, HE, and HA were involved in the study concept. JU, HE, EH, MC and HA were involved in the study design. DE, SJ, EH and MC performed data acquisition, JU, HE and HA were involved in data analysis/interpretation. JU, HE, DE, SJ, EH, MC and HA were involved in either manuscript preparation or editing. All authors read and approved the final manuscript.
